# Subjective memory complaints are associated with altered resting-state functional connectivity but not structural atrophy

**DOI:** 10.1016/j.nicl.2019.101675

**Published:** 2019-01-11

**Authors:** Toshikazu Kawagoe, Keiichi Onoda, Shuhei Yamaguchi

**Affiliations:** Department of Neurology, Faculty of Medicine, Shimane University, 89-1, Enya-cho, Izumo, Shimane 693-8501, Japan

**Keywords:** Aging, Subjective memory complaints, Resting-state fMRI, Voxel-based morphometry, Depression

## Abstract

Research indicates that a subtle cognitive decline, accompanied by pathological changes, occurs in individuals with subjective memory complaints (SMC). However, there is less evidence regarding the measurement of resting-state functional connectivity to detect subtle brain network alterations in neurodegenerative illnesses before cognitive change manifestation. We investigated the correlation between SMC and cognitive performance and explored functional and structural brain changes underlying SMC severity, using behavioral and brain imaging data-driven approaches. We observed that SMC was associated with depression but not with cognitive test scores, implying that SMC represent the “worried-well”; however, this model explains only 15% of the target variance. Using a conservative threshold, we observed connectivity related to SMC severity in the lingual gyrus, cuneus, anterior insula, and superior parietal lobule. Post-hoc analysis indicated that occipital and parietal functional connectivity increased with SMC severity. In contrast, volumetric alterations were not associated with SMC, even after applying a liberal threshold. Our findings suggest that altered resting-state functional connectivity in regions associated with SMC might reflect early compensatory changes that occur before cognitive and structural abnormalities develop.

## Highlights

• Subjective memory complaints are nearly independent from current cognition.
• Resting-state functional connectivity is related to subjective memory complaints.
• Brain structure has no association with subjective memory complaints.

## 1 Introduction

Recently, there has been growing interest in individuals with subjective memory complaints (SMC; also known as subjective cognitive impairment, subjective memory impairment, subjective cognitive decline, and other terminology) (Abdulrab and Heun, 2008) who experience changes in their memory function. Clinically, SMC may represent a prodromal state of mild cognitive impairment (MCI) (Dubois et al., 2016; Jessen et al., 2014; Petersen et al., 2001), although some studies have demonstrated that the cognitive state is not always related to actual detectable memory function (Harwood et al., 2004; Jungwirth et al., 2004; Kryscio et al., 2014). A meta-analysis indicated a significant association between SMC and actual memory function, although the effect size was small (Crumley et al., 2014). Such a small effect might be due to the fact that SMC represent subtle cognitive changes that fall below the detectable threshold of common cognitive tests. Indeed, recent studies with larger sample sizes have indicated that SMC at baseline can predict future memory decline related to dementia (Abner et al., 2015; Kaup et al., 2015; Koppara et al., 2015).

Interestingly, SMC appears to correlate with specific pathological processes related to MCI or Alzheimer's disease (AD). This includes protein markers in the cerebrospinal fluid, including amyloid β-42, total tau, and phosphorylated tau; structural patterns, identified with magnetic resonance imaging (MRI) or diffusion MRI; and functional brain states, identified with positron emission tomography or functional MRI (fMRI) (for a review, Sun et al., 2015). Indeed, structural changes in individuals with SMC have been observed, including reduced volume in medial regions, such as the entorhinal cortex, hippocampus, anterior cingulate, and precuneus (Hafkemeijer et al., 2013; Jessen et al., 2006), as well as cortical thinning in these regions (Schultz et al., 2015). However, the results of fMRI studies are somewhat controversial. During an episodic memory task, participants with SMC exhibited reduced hippocampal activation and increased prefrontal activation relative to control subjects (Erk et al., 2011). In contrast, another study showed increased activation in subcortical regions, including the hippocampus, in individuals with SMC performing a divided attention task (Rodda et al., 2011).

Previous studies have attempted to identify key brain regions associated with SMC using biochemical, structural, and functional methods. Such studies have confirmed that several brain regions play a role in SMC. However, most previous studies have used conventional model-dependent methods to examine outcomes in specific brain regions. More recently, functional neuroimaging data has been collected using resting-state fMRI (rs-fMRI) techniques, which can be used to measure spontaneous neural activity and to evaluate regional brain fluctuations occurring during rest or when a participant is not performing any explicit task (van den Heuvel and Hulshoff Pol, 2010). The resulting resting-state functional connectivity (rs-FC) correlates with the activity of distant brain regions. Measuring this allows the detection of subtle brain network alterations in neurodegenerative illnesses, such as AD, before the manifestation of cognitive and behavioral changes (Sheline and Raichle, 2013). Although rs-FC is an appropriate marker for the study of SMC, few studies have used rs-fMRI, albeit with inconsistent results. One study reported increased rs-FC within the default-mode network and medial visual network in individuals with SMC compared with control subjects (Hafkemeijer et al., 2013). The default-mode network is one of the large-scale brain networks that exhibits strong rs-FC, which is a key component of the aging brain (Sheline and Raichle, 2013). It consists of several brain regions, mainly including the posterior cingulate cortex, medial prefrontal cortex, and angular gyrus. This region is known to be involved in many functions, including autobiographical information, self-reference, theory of mind, social evaluations, episodic memory, etc. (Andrews-Hanna, 2012). However, another study indicated lower connectivity in the default-mode network in subjects with SMC when compared with control subjects, but higher connectivity than in individuals with MCI (Wang et al., 2013).

In the present study, we aimed to explore the relationship between SMC and rs-FC by using rs-fMRI. Although seed-based analysis provides a clear view of regions functionally connected with the seed region—and thus an elegant way of examining rs-FC in the human brain—the resulting information is limited to the functional connections of selected seed regions, making it difficult to examine connectivity patterns across the whole brain (van den Heuvel and Hulshoff Pol, 2010). Therefore, we implemented whole-brain multivariate pattern analysis (MVPA) for a data-driven, agnostic approach. The benefit of such an approach is that it circumvents the selection bias of using specific regions as nodes and is more reproducible than conventional seed-based approaches (Song et al., 2016). Additionally, we conducted voxel-based morphometry (VBM) (Ashburner and Friston, 2000) combined with structural brain MRI to examine whether the gray matter volume is associated with symptom severity and functional alterations in individuals with SMC.

Our objectives were: (1) to confirm the association between performance of cognitive functions and SMC in our dataset; (2) to explore brain regions in which rs-FC correlated with the SMC index; and (3) to investigate the relationship, if any, between functional and structural findings associated with SMC severity. Based on previous reports (Hafkemeijer et al., 2013; Jessen et al., 2006; Schultz et al., 2015; Cooley et al., 2015), we hypothesized that the rs-FC and volume of medial and posterior regions, such as the hippocampus, entorhinal, thalamus, posterior cingulate, fusiform, cuneus, and precuneus, which form part of the default-mode and visual networks, would be modulated by SMC severity. Finally, as a complementary analysis, we include follow-up data indicating that SMC are related to later cognitive decline.

## 2 Materials and methods

### 2.1 Participants

Participants were selected from the health examination system database at the Shimane Institute of Health Science (Kawagoe et al., 2017; Onoda et al., 2012). This database is a collection of medical, neurological, neuropsychological, MRI, and blood test data for individuals who underwent rs-fMRI scanning from December 2012 to September 2015. We included 322 older adults (60–94 years old), all of whom led independent lives in the community without any advanced medical treatment. They all voluntarily performed the above tests as a part of their health checkup, which included brain imaging and a cognitive function examination.

None of the participants expressed any severe complaints about their health at the visit, but participants were excluded if there was any suspicion of cognitive impairment [below the appropriate Mini-Mental State Examination (MMSE) cutoff], cerebral injuries, or abnormalities, such as any apparent atrophy, cerebral hemorrhage, or previous cerebral infarction, including silent infarction, brain edema, aneurysm, hypoplasia, empty sella syndrome, any type of cyst, enlarged perivascular space, hydrocephaly, chronic subdural hematoma, vessel malformation, marked periventricular hyperintensity (PVH), or leukoaraiosis. At least two specialists (radiologists and/or neurologists) confirmed these findings in addition to performing a standard neurological examination. If such abnormalities were below a moderate severity level, for example grade II for PVH (Shinohara et al., 2007), the individual was not excluded. We also excluded individuals with a medical history of cancer, heart disease, or severely decreased vision or hearing, as well as those with any history of cerebral disease, stroke, psychosis, or parkinsonism. Some participants were excluded because single or multiple data were missing. Therefore, the resulting sample included 155 participants (age: 69.5 [range: 60–83] years; 70 men and 85 women; demographic data are shown in Table 1). Just as a complementary analysis, we conducted a follow-up analysis. Of the 155 participants, only 14 (age: 69.2 [range: 60–77] years; 10 men) were included in this follow-up because our health examination system database does not have a study-first policy.

**Table 1:** Demographic and behavioral data for all participants (*N* = 155).

Variables	Mean	Standard deviation	Range
Age	69.56	5.60	60–83
Education	13.59	2.55	9–19
MMSE	29.38	0.74	28–30
SDS	34.71	7.63	21–65
AS	10.47	6.23	0–30
WCST_CA	4.70	1.15	1–6
WCST_PEN	3.17	2.85	0–14
WCST_DMS	0.71	1.01	0–4
FAB	16.63	1.18	12–18
KANA	41.37	10.65	14–59
VFT_‘vegetable’	15.72	3.75	7–24
VFT_‘*shi*’	9.30	3.21	1–19
SMS	31.99	4.59	16–40
OMS	7.12	2.89	1–14.5

MMSE, Mini-Mental State Examination; SDS, Self-rating depression scale; AS, apathy scale; WCST, Wisconsin card sorting task; CA, categories achieved; PEN, preservative errors of Nelson; DMS, difficulties of maintaining set; FAB, frontal assessment battery; KANA, *kanahiroi* test; VFT, verbal fluency test; SMS, subjective memory score; OMS, objective memory score.

The study was conducted in accordance with the Declaration of Helsinki (1975), as revised in 2008, and the regulations of the Japanese Ministry of Health, Labour and Welfare. The medical ethics committee of Shimane University approved the study. Written informed consent was obtained from all participants.

### 2.2 Neuropsychological and neuropsychiatric measures

All the participants underwent neuropsychological assessments, including the MMSE (Folstein et al., 1975) and frontal lobe/executive function tests: verbal fluency (Benton, 1968), frontal assessment battery (Dubois et al., 2000; Kugo et al., 2007), “Kanahiroi” test (Kaneko, 1990), and Wisconsin card sorting test (Anderson et al., 1991). Furthermore, they completed two psychiatric questionnaires: the self-rating depression scale (SDS; Zung et al., 1965) and apathy scale (AS; Starkstein et al., 1992) questionnaires.

In the verbal fluency tests, individuals were given one minute to generate names from a specified category (i.e., vegetables), the name of which started with a specific sound (/sa/ in Japanese). For the Wisconsin Card Sorting Test, we considered the number of “categories achieved,” “perseverative errors of Milner,” and the “difficulty maintaining set,” because these data could be obtained from the Keio Version that we used.

In the paper-based “Kanahiroi” test, participants identified and circled five kana letters (i.e., the Japanese vowels corresponding to A, E, I, O, and U) occurring in a story written in kana, which the participants read silently. The number of letters correctly identified in two minutes was recorded. The SDS and AS were used to detect depressive symptoms. SDS scores normally range from 20 to 80 and the AS scores range from 0 to 42, with higher scores indicating a more depressive or apathetic state, respectively.

The MMSE was used to initially screen for cognitive impairment with a cutoff score of 27/28, which is considered important to detect MCI. A recent meta-analysis suggested that this cutoff has a sensitivity of 66% and a specificity of 72% (Ciesielska et al., 2016). For the executive function tests, the scores were integrated into a single measure to avoid the problem of “task impurity” (Miyake et al., 2000; Shilling et al., 2002), which suggests that any score derived from an executive function task necessarily includes systematic variance attributed to non-executive-related function. For example, scores of the Wisconsin Card Sorting Test include assessment not only of executive function but also of color and figure processing speed, articulation speed, manual motor speed, etc. Unfortunately, this confounding variance is substantial and makes it difficult to clearly capture executive-related variance from a single test (Miyake and Friedman, 2012). Therefore, we unified scores from several tests for executive function into a single index by averaging *Z*-normalized values to minimize nuisance variance (Kawagoe et al., 2017; Reineberg et al., 2015). Nevertheless, we also analyzed our data for individual executive function tests to confirm the reliability of our results.

We extracted the results from the SMC questionnaire, as well as the “subjective memory score (SMS)” from our database. The SMS was calculated based on a previous questionnaire reported by Osada et al. (1997). The SMS questionnaire comprises 10 questions probing an individual's SMC. The questionnaire provides scores ranging from 10 to 40, with higher scores indicating less severe SMC. It reflects the degree of SMC as a continuous variable, which may provide better resolution than a dichotomized question. We also assessed the objective memory score (OMS) using an associated learning test from Okabe's Mini-Mental Scale, which is a modified verbal test included in the Wechsler Adult Intelligence Scale (Kobayashi et al., 1987). The objective memory test was administered by an experimenter who read out 10 pairs of words, half of which were semantically related and half of which were not. Participants were required to remember word pairs. After reading all the words, the experimenter read one word from each pair, and the participants responded with the paired word. This procedure was executed twice. Scores ranged from 0 to 15, with 0.5 points awarded for semantically-related word pairs and 1 point for unrelated pairs. Table 2 shows items of the OMS and SMS tests.

**Table 2:** List of items from (a) the subjective memory score questionnaire and (b) the associated learning test for objective memory scores.

a. Questionnaire for subjective memory score	
Read the statement and respond with the frequency that applies to you these days.	
1. When you look for something, you forget what you look for.	
2. You are forgetful of your promises.	
3. You cannot recall the name of your friends or relatives.	
4. You forget what you are about to say unexpectedly.	
5. You forget important days, such as a birthday or anniversary.	
6. You forget a deadline for a payment or promise.	
7. You experience tip-of-the-tongue phenomena.	
8. You forget where you put items for daily use such as glasses.	
9. You always lose something when you go somewhere.	
10. You forget to buy items when you have multiple items to buy.	
Choices: 1. Frequently 2. Sometimes 3. Occasionally 4. Never	
b. Items in the associated learning test for objective memory score	
1. Fruit and apple	6. Boy and *tatami*
2. Sky and the sun	7. Bud and tiger
3. House and yard	8. Rabbit and *shoji*
4. Travel and sights	9. Swimming and bank
5. Metal and iron	10. Bathing and assets

### 2.3 fMRI acquisition

Imaging data were acquired with a Siemens AG 1.5-T scanner (Symphony). We acquired 27 slices (each 4.5 mm thick) with no gap parallel to the plane connecting the anterior and posterior commissures with a T2*-weighted gradient-echo spiral pulse sequence [repetition time (TR) = 2000 ms, echo time (TE) = 30 ms, flip angle = 90°, interleaved order, matrix size = 64 × 64, field of view (FOV) = 256 × 256 mm^2^, and isotropic spatial resolution = 4 mm]. All participants were instructed to remain awake with their eyes closed as they underwent a 5-min rs-fMRI scan. After the functional scan, we obtained T1-weighted images of the entire brain (192 slices, TR = 2170 ms, TE = 3.93 ms, inversion time = 1100 ms, flip angle = 15°, matrix size = 256 × 256, FOV = 256 × 256 mm^2^, isotropic spatial resolution = 1 mm).

### 2.4 fMRI preprocessing

For preprocessing, we used the Statistical Parametric Mapping software (SPM12, http://www.fil.ion.ucl.ac.uk/spm) implemented in MATLAB R2017a (MathWorks, Natick, MA, USA). The images were realigned to remove any artifacts from head movements, with 6 parameters for bulk head motion (3 translations and 3 rotations) and 6 additional parameters that included the original derivatives. Furthermore, we corrected for differences in image acquisition time between slices. The realigned images were normalized to the Montreal Neurological Institute template standard space with a nonlinear warp transformation and resliced with a voxel size of 3 × 3 × 3 mm^3^. Spatial smoothing was then applied with a full-width at half-maximum (FWHM) of 6 mm.

Subsequently, the rs-FC temporal data were processed. The head movement time series, white matter signal, and cerebral spinal fluid signal were regressed out from each voxel in the first-level analysis. The data were bandpass filtered from 0.008 to 0.09 Hz and were linearly detrended and despiked in this step. Temporal processing and subsequent analyses were conducted using the functional connectivity toolbox 17.f (CONN; Whitfield-Gabrieli and Nieto-Castanon, 2012).

### 2.5 Statistical analyses

#### 2.5.1 Analyses for behavioral measures

We first conducted a multiple regression analysis for behavioral measures. This was calculated to predict the SMS based on other data, including age, sex, education, MMSE, SDS, AS, ZEF, and OMS, in a stepwise method (probability-of-F-to-enter ≤0.05; probability-of-F-to-remove ≥0.10). Before this regression, we tested the normality of each dataset, where appropriate, using the Kolmogorov–Smirnov test with Lilliefors significance correction. Based on the result, we determined which covariates required adjustment in the following analyses. In addition, for the complementary follow-up, correlation coefficients were calculated. The variables included the baseline SMC score and variability of the integrated score of executive function (ZEF) (follow-up score – baseline score).

#### 2.5.2 Multivariate pattern analysis for functional connectivity

**Fig. 1:**
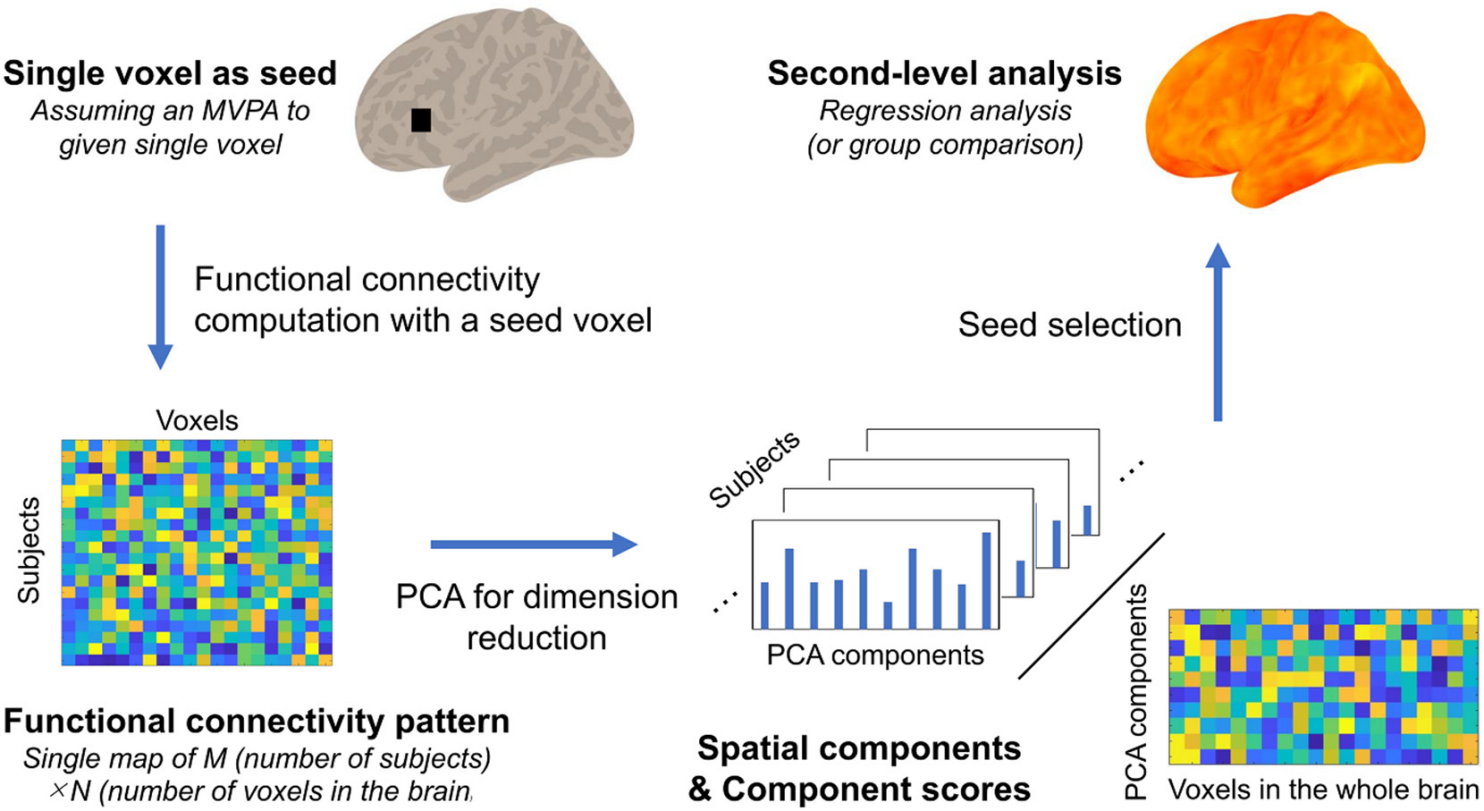
Illustration of resting-state multivariate pattern analysis (MVPA) procedure for a single voxel. First, a single voxel was seeded, and pairwise correlation patterns to all other voxels in the brain were calculated. Second, principle component analysis reduced the dimensions into an appropriate number of components (e.g., 10% of the number of samples) while maximizing inter-participant variability in the resulting correlation patterns. Subsequently, the spatial map and component scores were calculated. Next, we performed multivariate analyses to identify associations between any resulting component scores and subjective memory scores for each participant. In the actual MVPA, this process was effectively repeated for every voxel in the brain.

We performed a principle component MVPA, i.e., a “connectome MVPA” (Fig. 1). This analysis provides a regionally unbiased mapping of brain areas with whole brain connectivity patterns, as predicted by target variables (Whitfield-Gabrieli et al., 2016). In comparison to univariate models, MVPA takes the joint information of all features into account, as opposed to considering the features as independent from one another. The target variable here was the SMS. Specifically, pairwise connectivity patterns among all voxels in the brain were calculated separately for each voxel. The connectivity matrix of each participant was concatenated for all participants into a matrix of M (number of participants) x N (number of voxels in the brain) for each single voxel. The dimensions of these multivariate patterns were then reduced with principal component analysis, which maximizes the proportion of inter-participant variance explained by fewer components. This process produced a matrix of M (number of participants) x C (appropriate number of components). The number of components was determined according to the general rule of proportion (i.e., 10%) against the number of participants, as previously described (Nieto-Castanon, 2015). As a result, the principle component could explain 90.7% of the data, on average, for each voxel. Thus, the resulting component score accurately represented the whole brain connectivity pattern for each participant.

Next, we performed a second-level analysis, which consisted of an omnibus F-test to identify the main effect of variables of interest. Therefore, post-hoc general linear model (GLM) analyses were required to determine specific connectivity patterns in data associated with the degree of SMC. We created regions of interest (ROI) based on peak clusters from the MVPA results to further explore the rs-FC of these regions. To create ROIs, the height threshold was set at p < 0.005 [cluster-level of p < 0.05 with false discovery rate (FDR) correction]. Except for ROI creation, we applied conservative thresholds for all imaging analyses and set the uncorrected height threshold at p < 0.001 and the FDR-corrected cluster-level threshold at p < 0.05. According to the multiple regression analysis results, we included SDS and AS scores as covariates for the MVPA. Age was covaried out, as it is known to be a factor that strongly affects the rs-FC (Onoda et al., 2012). To test if head movement during scanning affected the results—although this nuisance was denoised at preprocessing—we calculated the correlation coefficients between the SMS and motion parameters (mean and maximum value for each of the six bulk motions). The results indicated that the relationship was too small, especially after the covariates (SDS, AS, and age) were regressed out (*r*s < 0.09).

#### 2.5.3 Voxel-based morphometry for structural data

Gray matter differences were assessed with VBM, as implemented in SPM12. The procedure consisted of two steps: preprocessing and statistical analysis. Preprocessing included gray matter segmentation, normalization with Diffeomorphic Anatomical Registration Through Exponentiated Lie algebra (DARTEL; Ashburner, 2007), and smoothing. At this step, each individual's T1-weighted image was denoised and segmented into gray matter, white matter, and cerebrospinal fluid on the basis of an algorithm in SPM12. An original DARTEL template for the total sample was created with the affine and DARTEL-warped gray matter, to attempt a closer match between the template and individual images. Each gray matter image was morphed into the original DARTEL template. Subsequently, modulated gray matter segments were determined with an 8-mm FWHM Gaussian kernel. In the statistical analysis, the smoothed gray matter segments were entered into a multiple regression analysis based on the GLM to explore regions in which volume correlated with SMC severity. Because the total brain volume could affect the results, especially in older adults, this variance was covaried out via analysis of covariance. As in the functional analyses, we used an uncorrected height threshold of p < 0.001 and an FDR-corrected cluster-level threshold of p < 0.05.

As with the rs-fMRI data, in addition to a mass-univariate approach, we also applied MVPA to the structural data for VBM analysis using the Pattern Recognition for Neuroimaging Toolbox (PRoNTo; Schrouff et al., 2013). We used the learned function called “*regression model”* and its predicting continuous measure (i.e. SMS score). The dataset (i.e. DARTEL-normalized gray matter images) was divided into training and test sets, and the analysis was partitioned into training and test phases. During the training phase, the algorithm learns some mapping between patterns and the labels on the training set. Then, during the test phase, the learned function is utilized to predict the SMS from data of the test set. Because a leave-one-out cross-validation approach was followed in this study, the training test process was repeated by the number of participants—namely the data of every participant were selected once as the test data, and the remaining participants' results were used as the training data. For the machine learning technique, Kernel Ridge Regression (Shawe-Taylor and Cristianini, 2004) was applied in PRonTo. This machine learning technique combines ridge regression (linear least squares with l2-norm regularization) with the kernel trick. Thus, it learns a linear function in the space, induced by the respective kernel and data. For validation, different metrics were used to compute the agreement between predicted and actual values, such as Pearson's correlation coefficient (*r*) and mean squared error (MSE). Permutation tests with 1000 repetitions were conducted to judge the significance of these metrics.

## 3 Results

### 3.1 Demographic and behavioral data

Table 1 presents the demographic and behavioral data for all participants. As described in Section 2.2, executive function test scores were integrated into a single score (ZEF) in the following analyses. One participant representing an outlier based on the SMS was excluded case-wise (smaller than mean + 3 standard deviations). None of the first-order correlations were above 0.70 (or below −0.70), and the highest squared correlation was 0.31, indicating that multicollinearity was not a problem in these data.

Because the original SMS result was negatively skewed, and the resulting residual was not normally distributed, we conducted the same analysis by using the log-transformed values for SMS (*new*X = LG10*[K–X]—where K is a constant from which the score is subtracted so that the smallest score is 1, and X is the original SMS), as the dependent variable (Howell, 2007). Due to this transformation, the polarity of SMS was reversed in this model (the higher the score the greater the severity). Although a significant regression equation was found [*F*(2, 151) = 16.42, p < 0.001], the model did not fit very well (R^2^ = 0.18, adjusted R^2^ = 0.17). The SMS was associated with the SDS (b* = 0.19) and AS (b* = 0.28) in our analysis, and the unstandardized residual normality was confirmed (*D* = 0.052, p = 0.20).

**Fig. 2:**
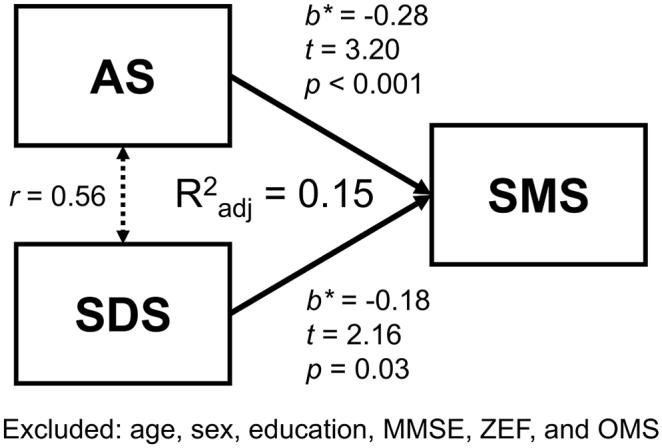
Results of the multiple regression analysis. In this model, the dependent variable was the original subjective memory score, which indicated that worse subjective memory complaints could be predicted by worse AS and SDS scores. AS: apathy score; SDS: self-rating depression scale; SMS: subjective memory score; MMSE: Mini-Mental State Examination; ZEF: integrated score of executive function; OMS: objective memory scale.

Because transformed data does not always accurately maintain the meaning of the original measurements (Grissom, 2000), we modeled the original SMS values via the same stepwise multiple linear regression analysis (Fig. 2). This suggested that SMS was significantly associated with the AS and SDS scores [*F*(1, 152) = 14.73, p < 0.001; R^2^ = 0.16, adjusted R^2^ = 0.15; SMS = constant – 0.28*AS – 0.18*SDS], although the residual was not normally distributed (*D* = 0.08, p = .017). In addition, the result remained unchanged even when scores for different executive function tests were modeled as independent variables [*F*(2, 151) = 16.42, p < 0.001; R^2^ = 0.15, adjusted R^2^ = 0.18; SMS = constant +0.28*AS +0.19*SDS]. Thus, the results did not differ much, regardless of whether the SMS were transformed and/or if the executive indices were integrated.

### 3.2 Functional neuroimaging data

Table 3a presents the MVPA results. As expected, three posterior regions (two of which were medial), the lingual gyrus, cuneus, and superior parietal lobule (SPL) showed rs-FC patterns with the whole brain and were associated with SMS. Connectivity patterns of the anterior insula also showed a significant association with SMS.

**Table 3:** Whole-brain multivariate pattern analysis and seed-based functional connectivity analysis.

Seed	Direction	Region	MNI coordinates	Voxel	Z
a. Multivariate pattern analysis					
N/A		R Lingual gyrus	[4, −60, 4]	20	4.07
		L Cuneus	[−10, −78, 24]	25	3.98
		L Anterior insula	[−34, 24, −10]	22	3.84
		L Superior parietal lobule	[−34, −44, 62]	18	3.77

b. Seed-based connectivity analyses (Regressor: SMS)					
R Lingual gyrus	Negative	R Precuneus R Calcarine R Cuneus	[6, −58, 10]	144	4.28
		L Cuneus	[−10, −78, 26]	83	4.13
		R Cuneus	[−10, −78, 26]	91	3.92
L Cuneus	Negative	L Precentral gyrus L Medial precentral gyrus	[−6, −28, 74]	168	4.96
		B Posterior cingulate gyrus L Lingual gyrus	[4, −42, 6]	98	4.75
		L Postcentral gyrus L Precentral gyrus	[−44, −18, 64]	110	4.56
		R Posterior cingulate cortex R Lingual gyrus	[18, −40, 4]	118	4.47
		R Fusiform gyrus R lingual gyrus	[22, −82, −12]	55	4.27
		B Lingual gyrus B Calcarine	[−12, −74, 0]	53	3.98
		R Postcentral gyrus	[14, −38, 78]	52	3.81
		B Precuneus	[2, −76, 46]	47	3.65
L Superior parietal lobule	Negative	R Superior parietal lobule	[24, −54, 68]	330	4.83
		L Superior parietal lobule L Postcentral gyrus	[−26, −48, 70]	90	4.02
		L Superior parietal lobule	[−30, −54, 58]	49	3.80

(a) Peak activation clusters based on whole-brain multivariate pattern analysis and regressed by the level of subjective memory complaints.

(b) Seed-based functional connectivity analyses. The association with subjective memory scores is depicted. Results of three analyses were thresholded by setting p < 0.05 with false detection rate-correction at the cluster level and uncorrected p < 0.001 at the peak level.

MNI: Montreal Neurological Institute; L: left; R: right; B: bilateral.

**Fig. 3:**
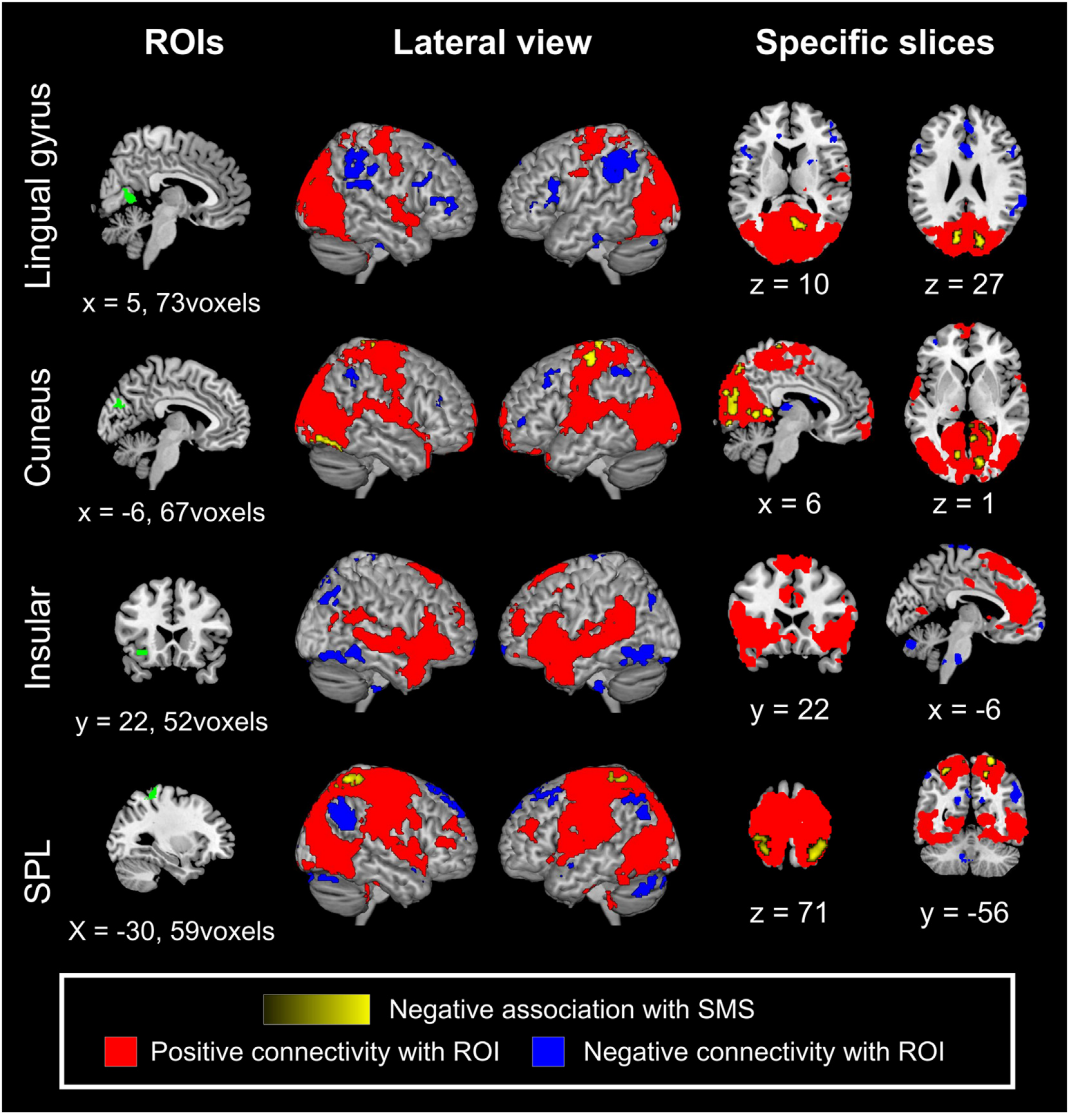
Resting-state functional connectivity associated with the level of subjective memory complaints when each region of interest was seeded.

Subsequently, we set these regions as separate ROIs to investigate rs-FC patterns associated with SMS. Simple functional connectivity is depicted in the blue and red binary maps of Fig. 3, and regions associated with SMS within simply connected areas are depicted in the yellow intensity maps of Fig. 3 and Table 3b. First, the lingual gyrus was functionally connected to the occipital and central brain areas. An association with SMS was confirmed in occipital regions, including the bilateral cuneus, right precuneus, and calcarine cortex. Second, the rs-FC of the cuneus was similar to that of the lingual gyrus, but it extended a bit further. An association with SMS was confirmed in the pre- and postcentral gyri, as well as in occipital visual regions. Third, the anterior insula was functionally connected to frontal and temporal regions, including the anterior cingulate cortex and bilateral anterior insula, which constitute the salience network (Seeley et al., 2007). However, we did not observe any associations with SMS within regions connected to this seed. This could be because regions whose connectivity with the insula correlated with the SMC level were not included in the area that was significantly and simply connected to the insula. Therefore, we did not focus on this connectivity pattern although an association in this region could have some implications. Finally, the SPL was simply connected with the temporal, parietal, and occipital cortices. When this was seeded, we confirmed an association with the SMS in the ipsilateral and contralateral SPL.

Collectively, four regions showed distinctive rs-FC patterns associated with SMC severity, independent of depression and apathy. Except for the anterior insula, the results indicated that more severe SMC were associated with stronger rs-FC at each seed (i.e., individuals with greater SMC had stronger rs-FC within occipital regions and between the ipsi- and contralateral SPL). Moreover, we seeded these three regions to explore OMS-related rs-FC. However, no regions showed significant associations with OMS, even when each ROI was seeded.

### 3.3 Structural imaging data

We implemented VBM analysis to investigate the relationship between SMC and brain volume. However, unlike our functional connectivity data, in the mass-univariate analysis, no associations were observed between the SMS and the volume of any region across the whole brain, even when we set a very liberal threshold (cluster-level uncorrected p < 0.001). Furthermore, even the MVPA failed to detect any relationship between SMC and brain structure. The *r* between real and predicted values was −0.09 and the MSE was 28.9. Permutation tests revealed that these values were non-significant (p = 0.73 and 0.52, respectively).

### 3.4 Complementary longitudinal analysis

We also conducted a complementary longitudinal analysis to confirm the association between SMC severity and future cognitive decline, although the sample size was very small (*n* = 14, approximately 10% of the total sample). The results indicated a significant association between baseline SMS and follow-up ZEF (*r* = −0.55, p = 0.040), even after controlling for the follow-up interval and age (*r*_p_ = −0.74, p = 0.009), suggesting that SMC represents a very early indication of cognitive decline.

## 4 Discussion

SMC refer to self-reported memory problems in daily life. Although it is controversial whether SMC are associated with objective cognitive deterioration, as some representative diagnostic criteria include this variable as evidence for MCI or preclinical AD (Dubois et al., 2016; Jessen et al., 2014; Petersen et al., 2001). Several studies have reported links between SMC and brain biochemistry, structure, and function (Sun et al., 2015; Hafkemeijer et al., 2013; Jessen et al., 2006). We aimed to replicate and extend previous findings showing an association between cognitive performance by exploring brain regions in which the rs-FC correlates with the SMC index.

By definition, SMC must represent self-experienced persistent decline in cognitive capacity, in comparison with a previously normal status (Jessen et al., 2014). According to this rigorous definition, perhaps our volunteer samples and questionnaire were not sufficient, and we should have dichotomized normal and pathological aging. However, previous SMC studies have used questionnaires similar to ours for assessing SMC in healthy older adults (Jungwirth et al., 2004; Schultz et al., 2015; Steinberg et al., 2013). With regard to the current topic, we argue that distinguishing normal and pathological aging is not suitable because the participants suspected for this condition would individually appear as “cognitively normal,” as the external test would not be able to detect the condition.

### 4.1 Behavioral associations with SMC

Our multiple regression analyses for behavioral data showed that, with the exception of depression and apathy, the severity of SMC was not associated with demographic and behavioral indices (Fig. 2). This result was not ascribed to the integration of indices of executive function and/or transformation of data. Indeed, we demonstrated that the level of SMC was not associated with the level of cognition, at least regarding executive function. However, our additional longitudinal analysis supports the notion that future cognitive decline might be reflected by the current SMC, although these results have to be viewed with caution due to the small sample size and possible bias in subject selection. Nevertheless, the results imply that SMC are a very early neuropsychological observation preceding cognitive deterioration.

Depression and apathy are significantly associated with the level of SMC. Although depression and apathy are sometimes discussed as distinct components (Landes et al., 2001; Levy et al., 1998), there is substantial overlap in their symptoms, such as diminished interest, psychomotor retardation, fatigue/hypersomnia, and lack of insight (Pagonabarraga et al., 2015). Therefore, we did not consider them as distinct components in this study. Previous studies have shown that negative emotions, such as depressive symptoms, are related to SMC severity rather than actual neuropsychological performance (Jungwirth et al., 2004; Crumley et al., 2014; Alegret et al., 2015). We have replicated these results in our dataset using the SMS, a continuous measure of SMC. Such findings might suggest that participants who report SMC are a “worried-well” population affected by their negative personality or emotional state. Alternatively, however, the SMS might actually identify initial subtle changes that fall below the detection thresholds of any of the cognitive tests, as the multiple regression model which included current widely-available neuropsychological assessments, could explain only 17% of the SMS variance. Therefore, we also consider that SMS might capture a feature that is largely independent from other variables, possibly representing a marker of early cognitive changes in older adults (Crumley et al., 2014; Abner et al., 2015; Kaup et al., 2015; Koppara et al., 2015).

### 4.2 Neural underpinnings of SMC

The interpretation of our behavioral results can be made more clear if we consider the neuroimaging data. After controlling for AS and SDS scores, MVPA indicated that four clusters in the brain were associated with SMS, according to their multivariate rs-FC pattern with other voxels in the brain. In contrast, no structural differences were associated with the degree of SMC, even using a liberal threshold.

#### 4.2.1 Brain function

Our main finding was that participants with higher level of SMC show stronger rs-FC within occipital regions seeded at the lingual gyrus and cuneus. The occipital area is a region that shows relative resistance to atrophic changes (Risacher et al., 2009). However, a previous study reported hypoperfusion in the occipital lobe in individuals with clinically defined MCI (Ding et al., 2014), and a recent previous meta-analysis demonstrated that activation (amplitude of low-frequency fluctuations) in the cuneus decreases in association with the level of cognitive impairment (Pan et al., 2017). These results suggest that functional alterations in individuals with MCI may be observed in this area. This is partly supported by behavioral evidence demonstrating the early decline of visual functions in older adults. For example, in independently-living older adults, including those with MCI, working memory capacity tends to degrade in the visual domain rather than the verbal domain (Kawagoe and Sekiyama, 2014; Kumar and Priyadarshi, 2013). Also, visual perceptual capacity is affected in the initial stages of the AD continuum (e.g., MCI) (Bublak et al., 2011). Against such decline, a previous study also reported that individuals in very initial stages (amyloid-negative) of MCI show hypermetabolism, predominantly in the occipital cortex, which might reflect a compensatory response to neural damage occurring early in the neurodegenerative process (Ashraf et al., 2015). Thus, we consider that our rs-FC results might reflect such a compensatory mechanism. This is consistent with a previous study reporting increased rs-FC in the medial visual network in older adults with SMC (Hafkemeijer et al., 2013). Therefore, although previous reports have shown inconsistency regarding the direction of activation/metabolism in the occipital area, we speculate that this region is functionally important during the very early stages of cognitive decline.

As in the case of the occipital visual area, we found that connectivity in the interhemispheric SPL is associated with the degree of SMC. The SPL is part of the parietal association area involved in several higher-order brain functions. The parietal region is uniquely affected in the early stages of AD (Geldmacher, 2003; Salimi et al., 2018) and plays a compensatory functional role in pre-symptomatic neurodegenerative diseases (Kloppel et al., 2009). Thus, interhemispheric SPL connectivity might also play a compensatory role in the initial stages of memory decline. Given that visuospatial function is affected early in the continuum of AD, and its dysfunction constitutes an accurate diagnostic clue (Salimi et al., 2018), brain regions related to this function may undergo changes in the initial stages of cognitive decline that are related to pathological changes. Therefore, the increased connectivity within/between occipital and parietal regions may represent an early physiological alteration resulting from neurodegenerative processes.

#### 4.2.2 Brain structure

In contrast to brain function, our results clearly demonstrate that brain structure is not associated with SMC severity. This was surprising because previous studies have indicated structural differences between individuals with SMC and healthy controls (Hafkemeijer et al., 2013; Jessen et al., 2006; Schultz et al., 2015). This discrepancy might be due to the cognition threshold that was set during the selection of participants. Unlike the threshold set in previous studies (Hafkemeijer et al., 2013; Schultz et al., 2015), we recruited participants who were leading independent lives and were considered to be “healthy” based on their health checkup. Because of this, our sample might have not yet exhibited any cognitive decline. In addition, there are differences in the analysis methods used. Our study used whole-brain regression analysis, while previous studies have used group comparisons and/or seed-based analysis (Jessen et al., 2006; Schultz et al., 2015). Therefore, we did not find any association between SMC and structural data in our population. Moreover, we captured the level of SMC as a continuous variate. Categorization simplifies the interpretation or presentation of results, but it might ignore some information and increases the risk of false positive results (Altman and Royston, 2006). Although our analysis might have a greater risk of false negative results and considering the relatively large sample size, the absence of SMS-associated regions using even a very liberal threshold (uncorrected, p < 0.001), and despite the fact that both univariate analysis and MVPA did not show any significant associations, we argue that functional alterations should precede structural brain changes.

#### 4.2.3 Aging effect

Although we covaried out the factor of age in all analyses, as it strongly affects cognition, SMC, functional connectivity, and structural integrity (Kumar and Priyadarshi, 2013; Onoda et al., 2012; Salthouse, 2009), its effect might have remained in the imaging analyses. To test this, we directly investigated the effect of age on functional and structural imaging data. We found that connectivity in many regions was associated with age. However, there was no overlap with the results of the MVPA, in which the target variables were SMC with SDS, AS, and age covaried out. With regard to the structural data, we also found a significant relationship with age, and several brain regions were associated with age; though, our original analysis did not find any association with SMC. Considering these results, we conclude that the effect of age was correctly covaried out from the original analyses.

### 4.3 Limitations

Some limitation should be considered when interpreting our results. First, we employed only one type of OMS to assess memory function, based on the verbal test from the Wechsler Adult Intelligence Scale. Using this test, we assessed only verbal short-term associative memory, but not principal memory function in daily life. The lack of tests to assess a wider range of memory functions might explain why the SMS was almost an independent variable in this study. Second, we did not directly assess if the participants expressed SMC, but instead we deduced it from their answers to the questionnaire. Because this procedure might have not reflected reality in their daily life, we should consider combining our results with those of previous studies, in which participants were divided as those with and those without SMC. As mentioned above, we found several changes in brain function associated with SMC. However, these were not associated with the default-mode network, which might be the most important region in the aging brain (Sheline and Raichle, 2013; Wang et al., 2013). This result could also be due to the continuous measurement of SMC.

### 4.4 Conclusions

SMC may represent a very early psychological indication of pathological cognitive decline. In this study, we demonstrated that SMC are independent from general cognitive functions and brain structure in healthy older adults but associated with the rs-FC within the occipital and parietal brain regions. SMC may constitute a useful marker for preclinical neurodegenerative diseases, such as MCI and AD. Further longitudinal studies are necessary to confirm the reliability and adequacy of SMC for screening.

## Compliance with ethical standards

The study was conducted in accordance with the Declaration of Helsinki (1975, as revised in 2008) and the regulations of the Japanese Ministry of Health, Labour and Welfare. The Shimane University medical ethics committee approved this study. Written informed consent was obtained from all participants. This work was supported by the Impulsing Paradigm Change through Disruptive Technologies Program (ImPACT) by a cabinet office in the government of Japan. All authors declare no conflicts of interest.

## References

[bb0005] Abdulrab K., Heun R. (2008). Subjective memory impairment. A review of its definitions indicates the need for a comprehensive set of standardised and validated criteria. Eur. Psychiatry.

[bb0010] Abner E.L., Kryscio R.J., Caban-Holt A.M., Schmitt F.A. (2015). Baseline subjective memory complaints associate with increased risk of incident dementia: the PREADVISE trial. J. Prev. Alzheimers Dis..

[bb0015] Alegret M., Rodriguez O., Espinosa A., Ortega G., Sanabria A., Valero S. (2015). Concordance between subjective and objective memory impairment in volunteer subjects. J. Alzheimers Dis..

[bb0020] Altman D.G., Royston P. (2006). The cost of dichotomising continuous variables. Br. Med. J..

[bb0025] Anderson S.W., Damasio H., Jones R.D., Tranel D. (1991). Wisconsin Card sorting Test performance as a measure of frontal lobe damage. J. Clin. Exp. Neuropsychol..

[bb0030] Andrews-Hanna J.R. (2012). The brain's default network and its adaptive role in internal mentation. Neuroscientist.

[bb0035] Ashburner J. (2007). A fast diffeomorphic image registration algorithm. NeuroImage.

[bb0040] Ashburner J., Friston K.J. (2000). Voxel-based morphometry-the methods. NeuroImage.

[bb0045] Ashraf A., Fan Z., Brooks D.J., Edison P. (2015). Cortical hypermetabolism in MCI subjects: a compensatory mechanism?. Eur. J. Nucl. Med. Mol. Imaging.

[bb0050] Benton A.L. (1968). Differential behavioral effects in frontal lobe disease. Neuropsychologia.

[bb0055] Bublak P., Redel P., Sorg C., Kurz A., Forstl H., Muller H.J. (2011). Staged decline of visual processing capacity in mild cognitive impairment and Alzheimer's disease. Neurobiol. Aging.

[bb0060] Ciesielska N., Sokolowski R., Mazur E., Podhorecka M., Polak-Szabela A., Kedziora-Kornatowska K. (2016). Is the Montreal Cognitive Assessment (MoCA) test better suited than the Mini-Mental State Examination (MMSE) in mild cognitive impairment (MCI) detection among people aged over 60? Meta-analysis. Psychiatr. Pol..

[bb0065] Cooley S.A., Cabeen R.P., Laidlaw D.H., Conturo T.E., Lane E.M., Heaps J.M. (2015). Posterior brain white matter abnormalities in older adults with probable mild cognitive impairment. J. Clin. Exp. Neuropsychol..

[bb0070] Crumley J.J., Stetler C.A., Horhota M. (2014). Examining the relationship between subjective and objective memory performance in older adults: a meta-analysis. Psychol. Aging.

[bb0075] Ding B., Ling H.W., Zhang Y., Huang J., Zhang H., Wang T. (2014). Pattern of cerebral hyperperfusion in Alzheimer's disease and amnestic mild cognitive impairment using voxel-based analysis of 3D arterial spin-labeling imaging: initial experience. Clin. Interv. Aging.

[bb0080] Dubois B., Slachevsky A., Litvan I., Pillon B. (2000). The FAB - a frontal assessment battery at bedside. Neurology.

[bb0085] Dubois B., Hampel H., Feldman H.H., Scheltens P., Aisen P., Andrieu S. (2016). Preclinical Alzheimer's disease: definition, natural history, and diagnostic criteria. Alzheimers Dement..

[bb0090] Erk S., Spottke A., Meisen A., Wagner M., Walter H., Jessen F. (2011). Evidence of neuronal compensation during episodic memory in subjective memory impairment. Arch. Gen. Psychiatry.

[bb0095] Folstein M.F., Folstein S.E., McHugh P.R. (1975). Mini-mental state. A practical method for grading the cognitive state of patients for the clinician. J. Psychiatr. Res..

[bb0100] Geldmacher D.S. (2003). Visuospatial dysfunction in the neurodegenerative diseases. Front. Biosci..

[bb0105] Grissom R.J. (2000). Heterogeneity of variance in clinical data. J. Consult. Clin. Psychol..

[bb0110] Hafkemeijer A., Altmann-Schneider I., Oleksik A.M., van de Wiel L., Middelkoop H.A., van Buchem M.A. (2013). Increased functional connectivity and brain atrophy in elderly with subjective memory complaints. Brain Connect.

[bb0115] Harwood D.G., Barker W.W., Ownby R.L., Mullan M., Duara R. (2004). No association between subjective memory complaints and apolipoprotein E genotype in cognitively intact elderly. Int. J. Geriatr. Psychiatry.

[bb0120] Howell D.C. (2007). Statistical Methods for Psychology.

[bb0125] Jessen F., Feyen L., Freymann K., Tepest R., Maier W., Heun R. (2006). Volume reduction of the entorhinal cortex in subjective memory impairment. Neurobiol. Aging.

[bb0130] Jessen F., Amariglio R.E., van Boxtel M., Breteler M., Ceccaldi M., Chetelat G. (2014). A conceptual framework for research on subjective cognitive decline in preclinical Alzheimer's disease. Alzheimers Dement..

[bb0135] Jungwirth S., Fischer P., Weissgram S., Kirchmeyr W., Bauer P., Tragl K.H. (2004). Subjective memory complaints and objective memory impairment in the Vienna-Transdanube aging community. J. Am. Geriatr. Soc..

[bb0140] Kaneko M. (1990). Dementia and frontal lobe function. Higher Brain Function Res..

[bb0145] Kaup A.R., Nettiksimmons J., LeBlanc E.S., Yaffe K. (2015). Memory complaints and risk of cognitive impairment after nearly 2 decades among older women. Neurology.

[bb0150] Kawagoe T., Sekiyama K. (2014). Visually encoded working memory is closely associated with mobility in older adults. Exp. Brain Res..

[bb0155] Kawagoe T., Onoda K., Yamaguchi S. (2017). Associations among executive function, cardiorespiratory fitness, and brain network properties in older adults. Sci. Rep..

[bb0160] Kloppel S., Draganski B., Siebner H.R., Tabrizi S.J., Weiller C., Frackowiak R.S. (2009). Functional compensation of motor function in pre-symptomatic Huntingtons disease. Brain.

[bb0165] Kobayashi S., Yamaguchi S., Kitani M., Okada K., Shimote K. (1987). Evaluation of practical usefulness of the Okabe's mini-mental scale in normal aged. Japanese J. Neuropsychol..

[bb0170] Koppara A., Wagner M., Lange C., Ernst A., Wiese B., König H.-H. (2015). Cognitive performance before and after the onset of subjective cognitive decline in old age. Alzheimers Dement. (Amst).

[bb0175] Kryscio R.J., Abner E.L., Cooper G.E., Fardo D.W., Jicha G.A., Nelson P.T. (2014). Self-reported memory complaints: implications from a longitudinal cohort with autopsies. Neurology.

[bb0180] Kugo A., Terada S., Ata T., Ido Y., Kado Y., Ishihara T. (2007). Japanese version of the frontal assessment battery for dementia. Psychiatry Res..

[bb0185] Kumar N., Priyadarshi B. (2013). Differential effect of aging on verbal and visuo-spatial working memory. Aging Dis..

[bb0190] Landes A.M., Sperry S.D., Strauss M.E., Geldmacher D.S. (2001). Apathy in Alzheimer's disease. J. Am. Geriatr. Soc..

[bb0195] Levy M.L., Cummings J.L., Fairbanks L.A., Masterman D., Miller B.L., Craig A.H. (1998). Apathy is not depression. J Neuropsychiatry Clin. Neurosci..

[bb0200] Miyake A., Friedman N.P. (2012). The nature and organization of Individual differences in executive functions: four general conclusions. Curr. Dir. Psychol. Sci..

[bb0205] Miyake A., Friedman N.P., Emerson M.J., Witzki A.H., Howerter A., Wager T.D. (2000). The unity and diversity of executive functions and their contributions to complex "Frontal Lobe" tasks: a latent variable analysis. Cogn. Psychol..

[bb0210] Onoda K., Ishihara M., Yamaguchi S. (2012). Decreased functional connectivity by aging is associated with cognitive decline. J. Cogn. Neurosci..

[bb0215] Osada Y., Shimonaka Y., Nakazato K., Kawaai C., Kikuchi Y. (1997). The development of self-rating scales in the elderly. Jpn. J. Gerontol..

[bb0220] Pagonabarraga J., Kulisevsky J., Strafella A.P., Krack P. (2015). Apathy in Parkinson's disease: clinical features, neural substrates, diagnosis, and treatment. Lancet Neurol..

[bb0225] Pan P., Zhu L., Yu T., Shi H., Zhang B., Qin R. (2017). Aberrant spontaneous low-frequency brain activity in amnestic mild cognitive impairment: a meta-analysis of resting-state fMRI studies. Ageing Res. Rev..

[bb0230] Petersen R.C., Doody R., Kurz A., Mohs R.C., Morris J.C., Rabins P.V. (2001). Current concepts in mild cognitive impairment. Arch. Neurol..

[bb0235] Reineberg A.E., Andrews-Hanna J.R., Depue B.E., Friedman N.P., Banich M.T. (2015). Resting-state networks predict individual differences in common and specific aspects of executive function. NeuroImage.

[bb0240] Risacher S.L., Saykin A.J., West J.D., Shen L., Firpi H.A., McDonald B.C. (2009). Baseline MRI predictors of conversion from MCI to probable AD in the ADNI cohort. Curr. Alzheimer Res..

[bb0245] Rodda J., Dannhauser T., Cutinha D.J., Shergill S.S., Walker Z. (2011). Subjective cognitive impairment: functional MRI during a divided attention task. Eur. Psychiatry.

[bb0250] Salimi S., Irish M., Foxe D., Hodges J.R., Piguet O., Burrell J.R. (2018). Can visuospatial measures improve the diagnosis of Alzheimer's disease?. Alzheimers Dement (Amst).

[bb0255] Salthouse T.A. (2009). When does age-related cognitive decline begin?. Neurobiol Aging.

[bb0260] Schrouff J., Rosa M.J., Rondina J.M., Marquand A.F., Chu C., Ashburner J. (2013). PRoNTo: pattern recognition for neuroimaging toolbox. Neuroinformatics.

[bb0265] Schultz S.A., Oh J.M., Koscik R.L., Dowling N.M., Gallagher C.L., Carlsson C.M. (2015). Subjective memory complaints, cortical thinning, and cognitive dysfunction in middle-aged adults at risk for AD. Alzheimers Dement..

[bb0270] Seeley W.W., Menon V., Schatzberg A.F., Keller J., Glover G.H., Kenna H. (2007). Dissociable intrinsic connectivity networks for salience processing and executive control. J. Neurosci..

[bb0275] Shawe-Taylor J., Cristianini N. (2004). Kernel Methods for Pattern Analysis.

[bb0280] Sheline Y.I., Raichle M.E. (2013). Resting state functional connectivity in preclinical Alzheimer's disease: a review. Biol. Psychiatry.

[bb0285] Shilling V.M., Chetwynd A., Rabbitt P.M. (2002). Individual inconsistency across measures of inhibition: an investigation of the construct validity of inhibition in older adults. Neuropsychologia.

[bb0290] Shinohara Y., Tohgi H., Hirai S., Terashi A., Fukuuchi Y., Yamaguchi T. (2007). Effect of the Ca antagonist nilvadipine on stroke occurrence or recurrence and extension of asymptomatic cerebral infarction in hypertensive patients with or without history of stroke (PICA Study). 1. Design and results at enrollment. Cerebrovasc. Dis..

[bb0295] Song X., Panych L.P., Chen N.K. (2016). Data-driven and predefined ROI-based quantification of long-term resting-state fMRI reproducibility. Brain Connect.

[bb0300] Starkstein S.E., Mayberg H.S., Preziosi T.J., Andrezejewski P., Leiguarda R., Robinson R.G. (1992). Reliability, validity, and clinical correlates of apathy in Parkinson's disease. J. Neuropsychiatry Clin. Neurosci..

[bb0305] Steinberg S.I., Negash S., Sammel M.D., Bogner H., Harel B.T., Livney M.G. (2013). Subjective memory complaints, cognitive performance, and psychological factors in healthy older adults. Am. J. Alzheimers Dis. Other Demen..

[bb0310] Sun Y., Yang F.C., Lin C.P., Han Y. (2015). Biochemical and neuroimaging studies in subjective cognitive decline: progress and perspectives. CNS Neurosci. Ther..

[bb0315] van den Heuvel M.P., Hulshoff Pol H.E. (2010). Exploring the brain network: a review on resting-state fMRI functional connectivity. Eur. Neuropsychopharmacol..

[bb0320] Wang Y., Risacher S.L., West J.D., McDonald B.C., Magee T.R., Farlow M.R. (2013). Altered default mode network connectivity in older adults with cognitive complaints and amnestic mild cognitive impairment. J. Alzheimers Dis..

[bb0325] Whitfield-Gabrieli S., Nieto-Castanon A. (2012). Conn: a functional connectivity toolbox for correlated and anticorrelated brain networks. Brain Connect.

[bb0330] Whitfield-Gabrieli S., Ghosh S.S., Nieto-Castanon A., Saygin Z., Doehrmann O., Chai X.J. (2016). Brain connectomics predict response to treatment in social anxiety disorder. Mol. Psychiatry.

[bb0335] Zung W.W., Richards C.B., Short M.J. (1965). Self-rating depression scale in an outpatient clinic. Further validation of the SDS. Arch. Gen. Psychiatry.

